# Cytoskeleton Protein EB3 Contributes to Dendritic Spines Enlargement and Enhances Their Resilience to Toxic Effects of Beta-Amyloid

**DOI:** 10.3390/ijms23042274

**Published:** 2022-02-18

**Authors:** Ekaterina Pchitskaya, Anastasiya Rakovskaya, Margarita Chigray, Ilya Bezprozvanny

**Affiliations:** 1 Laboratory of Molecular Neurodegeneration, Peter the Great St. Petersburg Polytechnic University, Khlopina St. 11, 194021 St. Petersburg, Russia; katrincreative@yandex.ru (E.P.); jonatepl@gmail.com (A.R.); margarita075@gmail.com (M.C.); 2 Department of Physiology, UT Southwestern Medical Center at Dallas, Dallas, TX 75390, USA

**Keywords:** dendritic spines, neuronal morphology, EB3, end-binding protein, PSD95, Synapsin CaMKII, neuroprotection, beta-amyloid, Alzheimer’s disease

## Abstract

EB3 protein is expressed abundantly in the nervous system and transiently enters the dendritic spines at the tip of the growing microtubule, which leads to spine enlargement. Nevertheless, the role of dynamic microtubules, and particularly EB3 protein, in synapse function is still elusive. By manipulating the EB3 expression level, we have shown that this protein is required for a normal dendritogenesis. Nonetheless, EB3 overexpression also reduces hippocampal neurons dendritic branching and total dendritic length. This effect likely occurs due to the speeding neuronal development cycle from dendrite outgrowth to the step when dendritic spines are forming. Implementing direct morphometric characterization of dendritic spines, we showed that EB3 overexpression leads to a dramatic increase in the dendritic spine head area. EB3 knockout oppositely reduces spine head area and increases spine neck length and spine neck/spine length ratio. The same effect is observed in conditions of amyloid-beta toxicity, modeling Alzheimer`s disease. Neck elongation is supposed to be a common detrimental effect on the spine’s shape, which makes them biochemically and electrically less connected to the dendrite. EB3 also potentiates the formation of presynaptic protein Synapsin clusters and CaMKII-alpha preferential localization in spines rather than in dendrites of hippocampal neurons, while its downregulation has an opposite effect and reduces the size of presynaptic protein clusters Synapsin and PSD95. EB3′s role in spine development and maturation determines its neuroprotective effect. EB3 overexpression makes dendritic spines resilient to amyloid-beta toxicity, restores altered PSD95 clustering, and reduces CaMKII-alpha localization in spines observed in this pathological state.

## 1. Introduction

The synapse is a contact between two neurons, serving to transmit information from one cell to another. Most synapses in the brain are formed between the axon of one neuron and the small membranous protrusion from the dendrite—the dendritic spine, of another neuron. Dendritic spines have various shapes and sizes, which are constantly changing in response to neuronal activity and part of synaptic plasticity mechanisms [1,2,3,4]. The vast majority of mature spines have a head and a thin neck, separating them from a dendrite [5,6]. Spine head size is proportional to the area of the postsynaptic density and the number of receptors at postsynapse [7,8,9,10], and therefore reflects the strength of a particular synapse. Spines with the biggest heads are called mushrooms and are thought to be the cellular sites of memory storage [5,11]. It is generally known that the dynamic cytoskeleton of dendritic spines consists of actin filaments [12,13] to enable rapid change of their shape, while microtubules (MTs) are cytoskeleton-organizing components in more stable parts of a neuron, such as axons and dendrites [14,15]. Recent studies revealed that dynamic microtubules, covered by end-binding protein 3 (EB3), enter dendritic spines; this process depends on neuronal activity [16,17,18,19,20] and leads to spines head enlargement [19]. The frequency of MTs’ entry into spines and the number of targeted spines increases after the induction of long-term potentiation (LTP) [19]. Thus, invasions of MTs into spines appear to be involved in the synaptic plasticity mechanisms and cargo transportation [21,22]. EB3 protein is expressed abundantly in the nervous system [16,17,18,19,20], but its role in synapse functioning is still elusive. Previously, it was shown that EB3 potentiates the formation of mushroom dendritic spines in hippocampal neurons and protects them from loss in the PS1-M146V-KI mice model of Alzheimer`s disease [18,23]. The reduction of mushroom spine fractions was shown in PS1-M146V-KI [24], APP-KI [25,26] mice models of Alzheimer`s disease (AD), in conditions of low amyloid toxicity [27], and elimination of mushroom spines has been proposed to underlie the memory loss observed in AD patients [28,29,30]. PS1-M146V-KI mice do not express human amyloid precursor protein (APP) nor generate human beta-amyloid (Aβ); therefore, they do not exhibit amyloid toxicity, which is thought to be the main driving force for AD. In this study, we tested EB3 neuroprotective potential in conditions of low-amyloid toxicity and investigated its role in the determination of dendrites and dendritic spine morphology using direct morphometric measurements. In addition, in this paper, we investigated EB3 expression-level influence on PSD-95, Synapsin protein clustering, and CaMKII-alpha localization. PSD-95 is the key scaffold protein of postsynaptic density in excitatory synapse dendritic spines, where receptors, kinases, other important proteins, and signaling molecules are densely packed to ensure effective signal receiving [31,32]. Immunocytochemistry with anti-PSD-95 antibodies is widely used to label postsynaptic density and investigate synaptic strength [33,34,35,36], since it is required for stabilizing and trafficking N-methyl-D-aspartic acid receptors (NMDARs) and α-amino-3-hydroxy-5-methyl-4-isox-azoleproprionic acid glutamate receptors (AMPARs) to the postsynaptic membrane [32,37]. Synapsin is the synaptic vesicle protein widely used for presynaptic contacts labeling and synapse density investigation [38]. CaMKII-alpha is a major synaptic kinase that is able to translocate to the stimulated spines and postsynaptic densities in response to neuronal activity [39]. Therefore, in this paper, the EB3 protein influence on hippocampal synapses at both pre- and postsynaptic sites using labeling with the most important synaptic proteins was investigated.

## 2. Results

### 2.1. Proper EB3 Expression Level Is Necessary for Normal Dendritogenesis of Hippocampal Neurons

**Figure 1:**
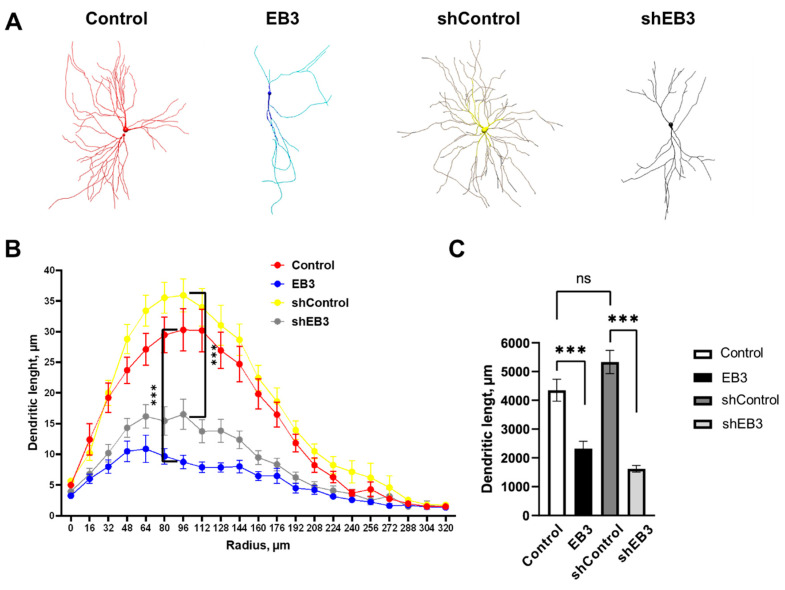
EB3 expression level affects the dendritogenesis of hippocampal neurons. (**A**) Models of hippocampal neurons transfected with mCherry or co-transfected with mCherry and EB3, plasmid encoding control RNAi (shControl) or RNAi against EB3 (shEB3) at DIV 7−8 and fixed at DIV 15 build in Neuronstudio from confocal images. (**B**) Dendritic length between two Sholl rings separated by 1 μm radius (distance from the soma) for each group of neurons. (**C**) The total length of dendrites for each group of neurons, μm. ***: *p* < 0.001, ns-no statistically significant difference, *n* = 20 neurons from 4 batches of cultures. Mann–Whitney U test for Sholl analysis, Student`s t-test for dendritic length, normal distribution was checked by the Shapiro-Wilk test.

It was shown that EB3 participates in the regulation of dendrite branching by association with PSD95 protein, which is thought to be the stop signal for dendrite branching [40]. Here we performed a comparative analysis of EB3 protein overexpression and downregulation on hippocampal neurons dendritogenesis. EB3 protein expression level effect on dendrites morphology was investigated in wild-type cultured hippocampal neurons in vitro. At seven days in vitro (DIV), when a neuron already has an axon and intensive dendrite branching is ongoing, the primary neuronal culture was transfected with a plasmid encoding a fluorescent protein mCherry (Control) or co-transfected with mCherry and plasmid encoding EB3, control RNAi (shControl) or RNAi against EB3 (shEB3). At DIV 15, the culture was fixed and then the series of confocal images were captured to visualize the morphology of the neurons. The model of neurons (Figure 1A) based on the 2D confocal images projection was built in Neuronstudio software and used for Sholl analysis (Figure 1B). The total dendritic length was measured between the two neighboring circles drawn from the center of neuron with the 1 um step difference in the diameter. Only some measurements are shown in Figure 1B to make it easier to read. Both EB3 overexpression and knockout lead to reduced dendrite branching (Figure 1B, ***: *p* < 0.0001) and total dendritic length (Figure 1C, ***: *p* < 0.0001). The total dendritic length was 4394.34 ± 380.88 µm in the control group, 5331.25 ± 405.79 μm in shControl group, 2327.50 ± 254.44 μm in EB3, and 1621.97 ± 116.86 μm in shEB3 groups, correspondingly.

### 2.2. EB3 Expression Level Controls Hippocampal Dendritic Spines Shape in Normal Conditions and in Conditions of Amyloid Toxicity

**Figure 2:**
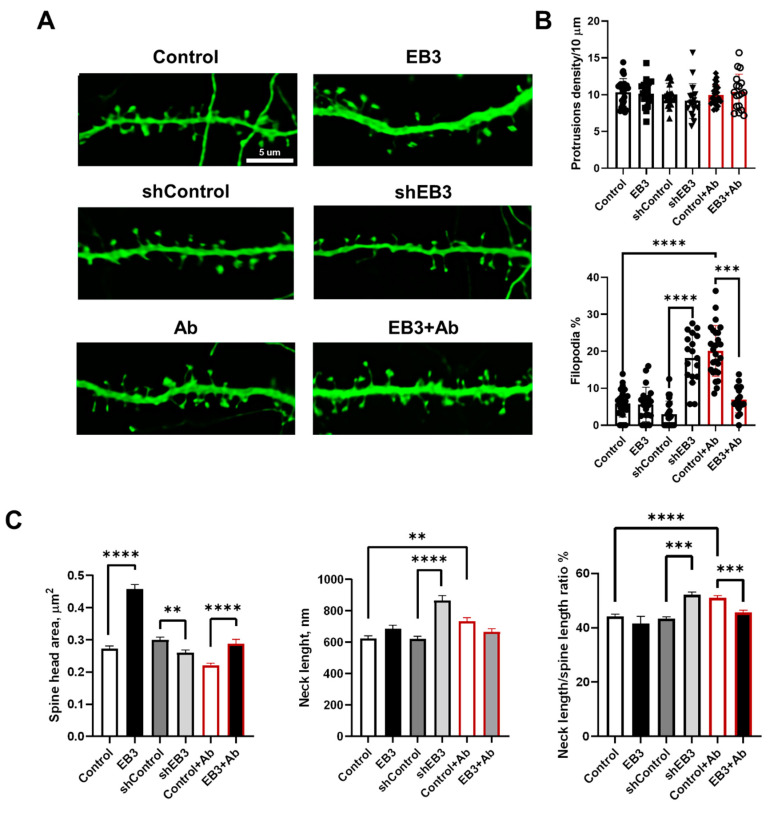
EB3 expression levels shape hippocampal dendritic spine morphology in WT neurons and in the condition of amyloid toxicity. (**A**) Confocal images of WT primary hippocampal dendrites and dendrites in the condition of amyloid toxicity (Ab) transfected with GFP or co-transfected with GFP and EB3, plasmid encoding control RNAi (shControl) or RNAi against EB3 (shEB3) at DIV 8-9, and fixed at DIV 16-17. Scale bar corresponds to 5μm. (**B**) The number of protrusions per 10 μm dendrite length and fraction of filopodia for each group of cells shown on panel A. (**C**) Direct morphometric measurements of dendritic spines morphology including spine head area, neck length, and neck length/spine length ratio. **: *p* < 0.01; ***: *p* < 0.001, ****: *p* < 0.0001. ANOVA test for protrusions density, Kruskal–Wallis test with post-hoc Dunn’s test for other data. *n* ≥ 18 neurons and *n* ≥ 230 spines from 3 batches of cultures.

In previous studies, we discovered that EB3 overexpression increases the percent of mushroom dendritic spines and protects them from elimination in hippocampal neurons derived from PS1-M146V-KI mice, modeling Alzheimer’s disease in vitro [23]. These transgenic mice do not have human amyloid precursor protein (APP) nor do they generate human Aβ, and therefore, lack amyloid toxicity, which is thought to be the main pathological hallmark of Alzheimer’s disease. Is the expression of EB3 protein able to protect dendritic spines from amyloid toxicity? To answer this question, we performed the analysis of hippocampal dendritic spines overexpressing EB3 after 72 h incubation with oligomeric Aβ42 fraction in low concentrations [27]. Another important question is the influence of EB3 expression levels on hippocampal dendritic spine shape. The authors and other groups previously showed that downregulation of EB3 leads to elimination of mushroom dendritic spines, while overexpression has the opposite effect [18,23]. Assessing the distribution of dendritic spines among predefined subtypes does not wholly reflect EB3′s role in shaping the spine’s morphology [11]. In order to investigate spine shapes, we co-transfected primary hippocampal neurons with GFP and mouse EB3 expression construct, shEB3, and shControl at DIV 7-8. At DIV 16-17 cultures were fixed, and analysis of dendritic spines morphology was performed by confocal microscopy (Figure 2A). 72 h before fixation, cells were treated with Aβ42 oligomers to model amyloid synaptotoxicity conditions in vitro. Then we performed direct measurement of morphological features describing dendritic spine shapes, including spine head area, neck length, and neck length/spine length ratio on 2D projections of dendritic images with newly developed SpineJ software [41]. This analysis was performed on spines with well-defined heads and necks without diving into thin or mushroom subtypes, since such spines present a continuum of spine shapes rather than distinct subtypes [2,11]. Stubby spines, which don’t have a neck to separate them from the dendrite, present only a minor fraction—less than 3–4%—of all the analyzed spines; therefore, we don’t include them in direct morphometric measurement analysis. Long spines without well-defined heads were labeled as filopodia, which presents a premature spine prototype. They also present a minor fraction—less than 4–5%—in WT neurons. Nevertheless, we analyzed the percent of filopodia due to the reason that shRNAi-based EB3 knockdown and amyloid toxicity leads to a significant increase in filopodia fraction. The density of dendritic protrusion was not significantly affected among all analyzed experimental groups (Figure 2B). In the shEB3 group and in neurons treated with oligomeric Ab, a fraction of filopodia-like protrusions significantly increased (Figure 2B) to 18.26 ± 1.59% (****: *p* < 0.0001) and 20.12 ± 1.35% (****: *p* < 0.0001), respectively. We may conclude that both conditions potentiate a formation of immature dendritic protrusions. EB3 overexpression significantly decreases the filopodia fraction to 6.945 ± 0.83% (***: *p* < 0.0001) to the level comparable with neurons in the control group. Spine head area is known to correlate with the area of the postsynaptic density (PSD), the number of postsynaptic receptors, and the ready-releasable pool of transmitter PSD area [10]. EB3 overexpression leads to a dramatic rise in spine head area, from 0.27 ± 0.01 μm^2^ to 0.46 ± 0.01 (****: *p* < 0.0001) μm^2^ in wild-type neurons and from 0.22 ± 0.01 μm^2^ to 0.28 ± 0.01 μm^2^ (****: *p* < 0.0001) in conditions of amyloid toxicity. In the EB3 knockdown group, spine head area drops to 0.26 ± 0.01, versus 0.30 ± 0.01 μm^2^ in the shControl group (**: *p* < 0.01). Spine neck length is tightly linked to its electrical properties, as well as the biochemical and electrical connection between the spine from its neighboring dendrite [10,42,43]. In the EB3 knockdown group, neck length increases to 865 ± 33 nm versus 621 ± 18 nm in the shControl group (****: *p* < 0.0001). Amyloid toxicity also leads to an increase in the spine neck length to 734 ± 20 nm, in comparison with 623 ± 18 in the control group (**: *p* < 0.01). Finally, the ratio between the neck length and the total spine length was analyzed in order to show what part of overall spine length falls on the neck. EB3 knockdown and amyloid toxicity leads to an increase in this ratio to 52.2 ± 0.91% (****: *p* < 0.0001) and 51.06 ± 0.72% (***: *p* < 0.001), in comparison to 44.20 ± 0.75 in the control group, and 43.28 ± 0.91 in the shControl group. EB3 overexpression in the condition of amyloid toxicity leads to a decrease in neck length/spine length ratio to normal levels (45.61 ± 0.83%, ***: *p* < 0.001).

### 2.3. EB3 Impacts on the Formation of PSD-95 and Synaptic Protein Clusters in Hippocampal Neurons in Normal Conditions and in Conditions of Amyloid Toxicity

**Figure 3:**
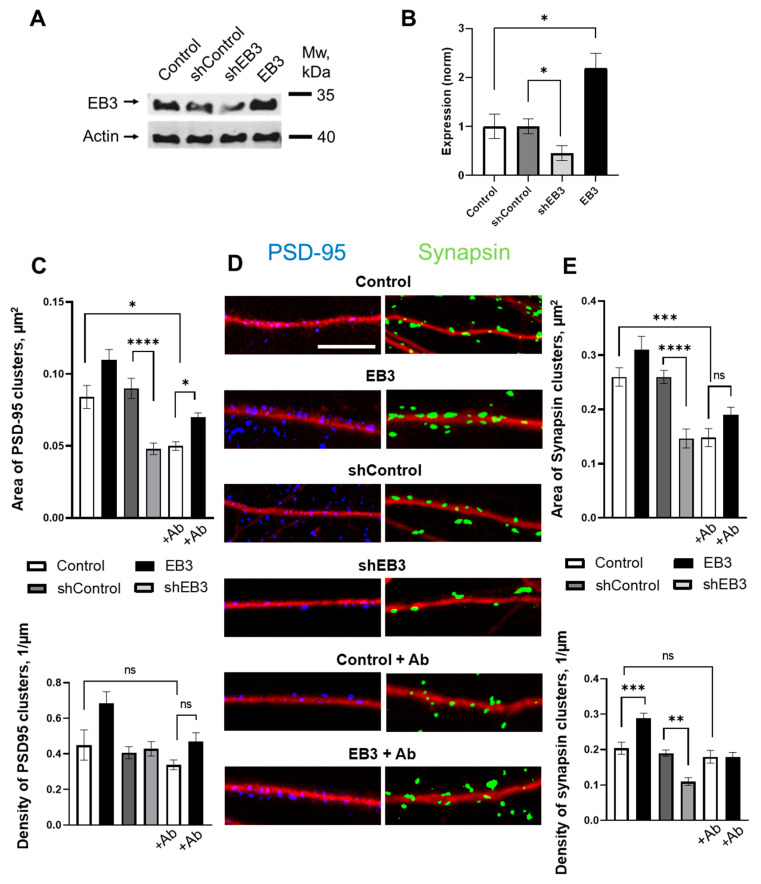
EB3 affects PSD-95 and Synapsin protein clustering. (**A**) The efficiency of EB3 expression level regulation generated in HEK293T cells Lentiviral particles were analyzed by Western blotting the primary hippocampal cultures infected with lentiviruses encoding EB3, control RNAi (shControl), or RNAi against EB3 (shEB3) at DIV 7-8 and lysed at DIV 15. Actin was used as a loading control. (**B**) Quantification of EB3 expression level (normalized to actin levels). The mean density of each band was normalized to an actin signal in the same sample and averaged. *n* = 4 batches of culture, *: *p* < 0.05. (**C**) Area and density of PSD-95 protein clusters for each group of cells shown on panel D. (**D**) Binarized confocal images of WT hippocampal dendrites and dendrites treated with beta-amyloid, infected with lentiviruses encoding EB3, control RNAi (shControl) or RNAi against EB3 (shEB3) at DIV 7 and fixed at DIV 14 and stained anti-MAP2 (red), anti-PSD95 (blue), and anti-Synapsin (green). Scale bar corresponds to 5μm. (**E**) Area and density of Synapsin protein clusters for each group of cells shown on panel D. *: *p* < 0.05, **: *p* < 0.01, ***: *p* < 0.001, ****: *p* < 0.0001, ns-no statistically significant difference. Tamhane’s T3 test for PSD95 clusters area, Kruskal–Wallis test with post-hoc Dunn’s test for other data. *n*  ≥  25 neurons from 3 to 5 batches of cultures.

To further study the effects of EB3 on neuronal maturation, we analyze the density and the size of PSD-95 and Synapsin 1 clusters, and postsynaptic and presynaptic proteins are shown to be robust general markers of synapses [44]. Cells were infected with lentiviruses encoding control RNAi (shControl), RNAi against EB3 (shEB3), or EB3 at DIV 7, and after fixation at DIV 14-16 neurons were subjected to IHC with antibodies against PSD95 (blue), Synapsin (green), and MAP2 (red) (Figure 3D). After binarization of the obtained confocal images area, density of PSD-95, and Synapsin protein, clusters were assessed. The control value for cluster densities was 0.31 ± 0.09 1/μm for PSD-95 and 0.21 ± 0.02 1/μm for Synapsin proteins (Figure 3C,E). Transduction of a neuronal culture with a control lentivirus carrying a random sequence not homologous to any of the cell messenger RNAs (shControl) did not affect the number and size of clusters. Virus-mediated overexpression of the EB3 protein has the trend to increase the size and density of PSD-95 clusters but has not reached significance, while the Synapsin clusters’ density is increased to 0.3 ± 0.01 1/μm. Knockdown of the EB3 protein leads to a decrease in PSD-95 protein clusters, sized from 0.090 ± 0.006 μm^2^ to 0.048 ± 0.004 μm^2^ (*p* < 0.0001), and Synapsin clusters from 0.260 ± 0.012 μm^2^ to 0.146 ± 0.017 μm^2^ (*p* < 0.0001), and a decrease in Synapsin cluster density from 0.19 ± 0.01 1/μm to 0.11 ± 0.01 1/μm (*p* < 0.01). For studies of the EB3 protein neuroprotective potential, oligomeric Aβ42 peptides were added for 72 h before the fixation. Conditions of low amyloid-beta toxicity don’t influence the PSD-95 and Synapsin clusters density in hippocampal neurons but cause a significant decrease in their size—areas of both protein clusters drop to the levels of 0.050 ± 0.003 μm^2^ (*p* < 0.0001) for PSD95 and 0.148 ± 0.017 μm^2^ (*p* < 0.0001) for Synapsin (Figure 3C,E). Virus-mediated overexpression of the EB3 protein increases PSD-95 cluster area to 0.070 ± 0.003 1/μm (*p* < 0.05) in conditions of amyloid toxicity.

### 2.4. EB3 Increases CaMKII-Alpha Spines to Dendrite Ratio in Normal Condition and in Conditions of Amyloid-Beta Toxicity

**Figure 4:**
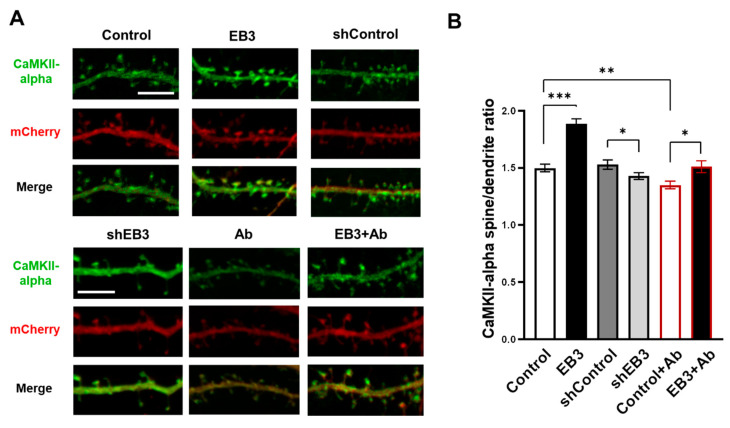
EB3 potentiates spine localization of CaMKII-alpha. (**A**) Confocal images of WT primary hippocampal dendrites and dendrites in condition of amyloid toxicity (Ab) co-transfected with Venus-CaMKII-alpha and mCherry infected with lentiviruses encoding EB3, control RNAi (shControl), or RNAi against EB3 (shEB3) at DIV 8-9 and fixed at DIV 16-17. Scale bar corresponds to 5μm. (**B**) The ratio of the CaMKII-alpha fluorescence intensity in spines to parent dendrite normalized to the mCherry. *: *p* < 0.05, **: *p* < 0.01, ***: *p* < 0.001. Student-t test. *n* ≥ 53 spines from 4 batches of cultures.

To investigate the localization of CaMKII-alpha, neurons were co-transfected with the plasmid Venus-CaMKII-alpha and mCherry and infected with lentiviruses encoding control RNAi (shControl), RNAi against EB3 (shEB3), or EB3 at DIV 8-9. At DIV 16-17, cultures were fixed, and analysis of fluorescent protein distribution was performed using confocal microscopy (Figure 4A). To assess the CaMKII-alpha spine/dendrite ratio we utilized the approach in [45,46,47]. Briefly, CaMKII intensity in spines and in a segment of the same area in the neighboring dendrite normalized to mCherry intensity, and then the ratio of the normalized CaMKII fluorescence intensity between the spine head and the adjacent dendrite region was calculated. CaMKII-alpha spine/dendrite ratio was 1.49 ± 0.04 in the control group and 1.53 ± 0.04 in the shControl group. Virus-mediated EB3 overexpression increased in the CaMKII-alpha spine/dendrite ratio to 1.88 ± 0.04, which indicates a predominantly synaptic localization of CaMKII (***: *p* < 0.001). Virus-mediated EB3 knockout and application of oligomeric beta-amyloid reduced the CaMKII-alpha spine/dendrite ratio to 1.43 ±0.03 and 1.35 ± 0.03, correspondingly (*: *p* < 0.05, **: *p* < 0.01). Virus-mediated EB3 overexpression restores the CaMKII-alpha spine/dendrite ratio in conditions of beta-amyloid toxicity to 1.51 ± 0.05 (*: *p* < 0.05).

## 3. Discussion

Based on the obtained data, we may conclude that EB3 protein level has a strong impact on both morphology of dendrites and dendritic spines (Figure 1). Previously it was shown that PSD-95 interacts with a proline-rich region of EB3 and alters microtubule dynamics and dendrite branching [40]. In this study we have shown that the proper EB3 protein level is crucial for normal dendritogenesis in hippocampal neurons (Figure 1). EB3 overexpression leads to a reduction of the dendrite branching and, at the same time, causes the formation of mature dendritic spines with large heads. Previously it was shown that during neuronal culture development, EB3 is expressed at relatively low levels in the early stages, and then its amount rapidly rises when a neuron enters the synaptic contacts maturation stage [18]. We may assume that EB3 overexpression, starting at DIV7, speeds the neuronal development cycle to spine formation; therefore, dendritic branching and outgrowth stops beforehand. Also, EB3 together with EB1 participate in the axon initial segment formation in neurons [48] and promote cilia formation in mouse fibroblasts [49]. Recent papers have also concluded that the loss of EB3 opposite to EB1 inhibited neuritogenesis in cortical neurons and concluded that drebrin/end-binding protein 3 (EB3) promotes linking F-actin to microtubules in filopodia [34]. It is likely that the same pathway may underlie EB3′s role in the spine maturation process. Taken together, EB proteins play an important role in the organization of various cell protrusions and extensions, and this is also confirmed for EB3 protein in hippocampal neurons.

We showed that EB3 overexpression leads to a dramatic increase in the dendritic spine head area. This is consistent with previous findings, indicating increases in mushroom dendritic spine fraction in hippocampal neurons during EB3 overexpression [18,23]. However, a more detailed impact of EB3 on spine morphology has not been investigated yet. Using direct morphometric measurements, we found out that EB3 knockout oppositely reduces spine head area and increases spine neck length and spine neck/spine length ratio (Figure 2). The same effect is observed in conditions of amyloid-beta toxicity, modeling Alzheimer’s disease in vitro. More and more studies confirm that spine neck geometry plays an important role in the determination of dendritic spine characteristics and plasticity [6,43,50]. Neck elongation is supposed to be a common detrimental effect on spine shape, which makes them biochemically and electrically less connected to the dendrite. Moreover, EB3 expression increases Synapsin protein cluster density, which is a widely used marker of presynapses, and CaMKII-alpha localization at spines (Figure 3 and Figure 4). CaMKII-alpha translocates to spines as a consequence of their activation [51,52,53,54,55]. EB3 knockout has a robust detrimental effect on both PSD-95 and Synapsin protein clustering. Taking everything into consideration, these results suggest that EB3 is involved in spine development and potentiates its maturation and formation of functional synaptic contacts.

In previous studies, we discovered that EB3 overexpression protects mushroom dendritic spines from elimination in primary hippocampal neurons derived from PS1-M146V-KI Alzheimer’s disease mouse model [23]. It this study, we evaluated EB3 neuroprotective potential in conditions of amyloid toxicity, which is thought to be the main driver of Alzheimer’s disease pathology. From the obtained data, including spine morphology and synaptic protein expression analysis, we may conclude that EB3 is able to protect dendritic spines from the toxic effects of beta-amyloid. Neuroprotective effects of this protein may be further investigated in order to develop a therapy for Alzheimer’s disease or other pathologies linked with synapse destabilization and loss.

## 4. Materials and Methods

### 4.1. Primary Hippocampal Cultures and Calcium Phosphate Transfection

Primary hippocampal neuronal cultures of dissociated hippocampal cells were prepared from newborn FVB mice. Briefly, the hippocampus of postnatal day 0–1 mouse pups were digested with papain solution (30 min at 37 °C; Worthington, OH, USA, #3176), then dissociated with 5 mg/mL Deoxyribonuclease I (Macherey Nagel GMBH, Germany, #R1542S) solution. Neurons were plated in a 24-well culture plate on 12 mm glass coverslips precoated with 1% poly-D-lysine (Sigma, USA, #p-7886) in Neurobasal-A (Gibco, UK, #10888022) medium supplemented with 2% B27 (Gibco, USA, #17504044), 1% heat-inactivated fetal bovine serum (FBS, Gibco, UK, #10500064), 0.5 mM L-Glutamine (Gibco, UK, #25030024), and maintained at 37 °C in a 5% CO2 incubator at 24-well glass plate. Transfection is performed at DIV 6-7, then at DIV 15-16, when hippocampal neurons reach maturity and form extensive synaptic contact cells, cells are fixed with a solution of 4% formaldehyde and 4% sucrose in PBS, pH 7.4 for 15 min and then extensively washed with PBS to remove fixation solution. Transfection was performed according to [41] with a calcium transfection kit purchased from Clontech (#631312). For transfection, plasmids pLV-mCherry (Clontech, USA, #632562), pLV-eGFP (Addgene, USA, #36083), Venus-CaMKII-alpha (Addgene, USA, #29427), FLAG-EB3 (described in [12]), control short hairpin RNA interference (shRNAi) (Sigma, USA, #SHC002), and mouse EB3–shRNAi (Sigma, USA, #SHCLNG-NM_133350, #TRCN0000315588) were used. Preparation of oligomeric beta-amyloid described in [27].

### 4.2. Lentiviral Particles Producing in HEK-293T Cells

HEK293T line cells with 50–70% of confluency were co-transfected with shuttle lenti-FLAG-EB3, lenti-shRNAi to scrambled RNA sequence (shControl) or against mouse EB3 (shEB3) and two helper plasmids pCMVΔ8.9 and pVSVg using polyethylenimine reagent (Polyscience, USA, #23966) in serum-free Opti-Mem medium (Gibco, UK, #11058-021). After 5–6 h incubation in a CO2 incubator, Opti-Mem was replaced with neuronal culture medium (Neurobasal A, 0.5 mM L-glutamine, 2% B27). 48–72 h after transfection, culture medium was collected, centrifuged 5 min at 2000 rpm, filtered through 0.45 um pore, immediately frozen in liquid nitrogen and then stored at −80 °C. Influence of each batch of generated lentiviruses on EB3 protein expression level in hippocampal neuronal culture was tested by Western blot with rabbit anti-EB3 antibodies (Abcam, UK, #ab157217) and mouse anti-actin (1:1000, Millipore, USA, #MAB1501) antibodies as loading control (Figure 3A,B). The amount of virus-containing medium with minimal toxicity and maximum infection efficiency was used in all experiments. 100–150 uL of lentivirus containing medium was added per well to hippocampal neuronal cultures at DIV 8 (next day after calcium–phosphate transfection).

### 4.3. Analysis of Dendritic Arborization and Dendritic Spine Morphology in Primary Hippocampal Cultures

For assessment of dendrites and dendritic spine morphology, a Z-stack of the optical section was captured with a confocal microscope (Thorlabs, USA). For dendritic analysis 2048 × 2048 pixel images with 0.25 μm/pixel resolution were captured with Z interval of 1 μm using a 20× objective lens (NA = 0.85, UPlanSApo; Olympus, Japan). For dendritic spine analysis, 2048 × 2048 pixel images with 0.025 μm/pixel resolution were captured with Z interval of 0.1 μm using a 100× objective lens (NA = 1.4, UPlanSApo; Olympus, Japan). Quantitative analysis of dendritic length and branching was performed with the help of the Sholl analysis module of a freely available Neurostudio software package [56]. Quantitative analysis of dendritic spines, including measurements of dendritic spine head area, neck length, and neck length/dendritic spine length ratio, was performed using SpineJ software [41]. Before analysis, dendritic protrusions in cultured primary hippocampal neurons were classified as headed spines, which have clearly defined head and neck, filopodia—An extremely long protrusions and stubby, relatively short protrusions without neck. Quantitative analysis was performed only on headed spines. At least 18 transfected neurons from three independent experiments were used for quantitative analysis.

### 4.4. PSD95/Synapsin Puncta Analysis

Fixed primary hippocampal neurons washed three times with PBS and permeabilized in 0.25% Triton X-100 in PBS solution for 15 min at room temperature. Nonspecific binding was blocked by incubating cells in 5% bovine serum albumin (BSA) in PBS solution for 1 h. Primary antibodies anti-MAP2 (1:1000, Milipore, USA, #MAB378), anti-synapsin I (1:1000, Chemicon, USA), and anti-PSD-95 (1:300, ThermoFisher, USA, #MA1-046) were diluted in 2.5% BSA in PBS and incubated at +4 °C overnight. After three washes, the hippocampal cultures were incubated in 2.5% BSA in PBS solution with the secondary antibody (1:1000, Invitrogen, USA, Alexa Fluor 488 #R37118 or 594 #R37121) for 1 h at room temperature and visualized with a confocal microscope. For PSD95/Synapsin puncta analysis, 2016 × 2016 pixels images with 0.034 μm/pixel resolution were captured with Z interval of 0.2 μm using a 100× objective lens (UPlanSApo, Olympus, Japan). All images were pre-processed with a rolling ball filter to reduce noise. The MAP2 images were binarized using the Lee minimum cross-entropy method to identify dendrite boundaries. PSD95/Synapsin images were binarized with ImageJ Adaptive 3D Threshold 1.22 plugin (http://www.pvv.org/~perchrh/imagej/thresholding.html) (accessed on 20 December 2021) with the following parameters: r = 2, C = 160, local weight = 5%, to identify protein clusters. Further, the density and area of the clusters were calculated using the Synpanal software [57].

### 4.5. CaMKII-Alpha Localization Analysis

To analyze CaMKII-alpha expression in spines, a 2048 × 2048 pixels Z-stack of mCherry and Venus-alpha was captured with a confocal microscope (Thorlabs, USA) with 0.03 μm/pixel resolution, and 0.1 Z interval using a 100× objective lens (NA = 1.4, UPlanSApo; Olympus, Japan). To assess the CaMKII-alpha spine/dendrite ratio we utilized the approach from [29,30,31]. Briefly, to compare the expression of CaMKII in spines and dendrites, mean gray value of CaMKII and Cherry intensities on 2D projection was measured in the segment corresponding to the spine head and in a segment of the same area in the neighboring dendrite using ImageJ software. The average CaMKII-alpha intensity was normalized to the mCherry in the same segment, and then the ratio of the normalized CaMKII-alpha fluorescence intensity between the spine head and the adjacent dendrite region was calculated.

### 4.6. Statistical Analysis

The statistics were analyzed using R Studio and Microsoft Excel programs. Statistical comparison of the data with normal distribution was carried out using the Student’s t-test and ANOVA. Normality was checked using the Shapiro–Wilk test. For the data with non-normal distribution nonparametric comparison Kruskal–Wallis test with post-hoc Dunn’s test was used. Statistical tests are indicated in figure legends. Data is presented as mean ± S.E.M.

## Data Availability

The data presented in this study are available in the article.
